# Broadband (550–1350 nm) diffuse optical characterization of thyroid chromophores

**DOI:** 10.1038/s41598-018-27684-8

**Published:** 2018-07-03

**Authors:** Sanathana Konugolu Venkata Sekar, Andrea Farina, Alberto Dalla Mora, Claus Lindner, Marco Pagliazzi, Mireia Mora, Gloria Aranda, Hamid Dehghani, Turgut Durduran, Paola Taroni, Antonio Pifferi

**Affiliations:** 1 Politecnico di Milano, Dipartimento di Fisica, Milano, Italy; 2 Consiglio Nazionale delle Ricerche, Istituto di Fotonica e Nanotecnologie, Milano, Italy; 3 ICFO-Institut de Ciències Fotòniques, Barcelona, Spain; 4 IDIBAPS, Fundació Clínic per la Recerca Biomèdica, 08036 Barcelona, Spain; 5 Hospital Clínic of Barcelona, Endocrinology and Nutrition Department, Barcelona, Spain; 6 University of Birmingham, School of Computer Science, Edgbaston, Birmingham, United Kingdom; 7 Institució Catalana de Recerca i Estudis Avançats (ICREA), 08015 Barcelona, Spain

## Abstract

Thyroid plays an important role in the endocrine system of the human body. Its characterization by diffuse optics can open new path ways in the non-invasive diagnosis of thyroid pathologies. Yet, the absorption spectra of tyrosine and thyroglobulin–key tissue constituents specific to the thyroid organ–in the visible to near infrared range are not fully available. Here, we present the optical characterization of tyrosine (powder), thyroglobulin (granular form) and iodine (aqueous solution) using a time domain broadband diffuse optical spectrometer in the 550–1350 nm range. Various systematic errors caused by physics of photo migration and sample inherent properties were effectively suppressed by means of advanced time domain diffuse optical methods. A brief comparison with various other known tissue constituents is presented, which reveals key spectral regions for the quantification of the thyroid absorbers in an *in vivo* scenario.

## Introduction

Thyroid hormone secretion plays a crucial role in the endocrine functions of the human body. Thyroid nodules are a common occurrence and recent studies have revealed that the incidence of thyroid cancer is rapidly increasing in modern society^1^. The study of thyroid nodular pathology is of great interest in the field of endocrinology given its high prevalence: palpable nodules are around 5%, but their prevalence reaches about 19–76% after cervical ultrasound^2^. Although the risk of malignancy is low (less than 5–10% of nodules)^3^, the high prevalence of these nodules requires a careful study to rule out malignancy. This study consists of performing a thyroid ultrasound and a fine needle aspiration biopsy (FNAB) of the nodule when appropriate. Ultrasound characteristics of the nodules can help us to distinguish between a benign and a malignant lesion. However, few features are specific of malignancy. Moreover, FNAB, that is a minimally invasive approach that can cause pain and discomfort, may have problems with sensitivity and specificity, with a 25% false negative and a 10% false positive rates^4^. These limitations lead to the search of complementary techniques to improve the specificity and, also, to exploit non-invasive methods to diagnose thyroid pathologies in order to reduce unnecessary invasive procedures.

In literature, various optical techniques have been explored to diagnose thyroid related pathologies, such as diffuse optical spectroscopy^5,6^, diffuse correlation spectroscopy^5^, static and time-resolved fluorescence spectroscopy^7,8^, optical biopsy using Raman spectroscopy^9,10^, Fourier transform infrared spectroscopy^11^, optical coherence tomography^12^, elastic light-scattering spectroscopy^13^ and two-photon excited fluorescence spectroscopy^14^. Among these techniques, diffuse optical approaches are non-invasive in nature and have the capability to investigate both chemical and functional properties of the organ. In recent decades, diffuse optics has been successfully applied to various human *in vivo* tissue studies: optical mammography^15,16^, tissue diagnosis^17^, optical biopsy of bone^18^, skin cancer diagnosis^19^, brain hemodynamics monitoring^20^ are few examples. Importantly, diffuse optics quantifies tissue constituents such as lipid, water, collagen, melanin, oxy- and deoxy-hemoglobin, by fitting the measured *in vivo* spectra to a library of tissue constituent spectra, which could be correlated to physiological and pathological conditions of the organ under study.

The diffuse optical study of thyroid is still in its nascent stage. Our previous work, Lindner *et al*.^5^, showed interesting variations in the hemodynamics of the healthy and nodular thyroid tissue, thus opening further interests in the diffuse optical community. However, this study–due to the limited number of wavelengths–considered only oxy- and deoxy-hemoglobin, neglecting the other tissue constituents that are specific to the thyroid organ and may have significant contribution in the near-infrared. This, in turn, may have caused some systematic artifacts in the absolute quantification, and may also imply that potential biomarkers that are accessible to the technique are not exploited.

**Figure 1:**
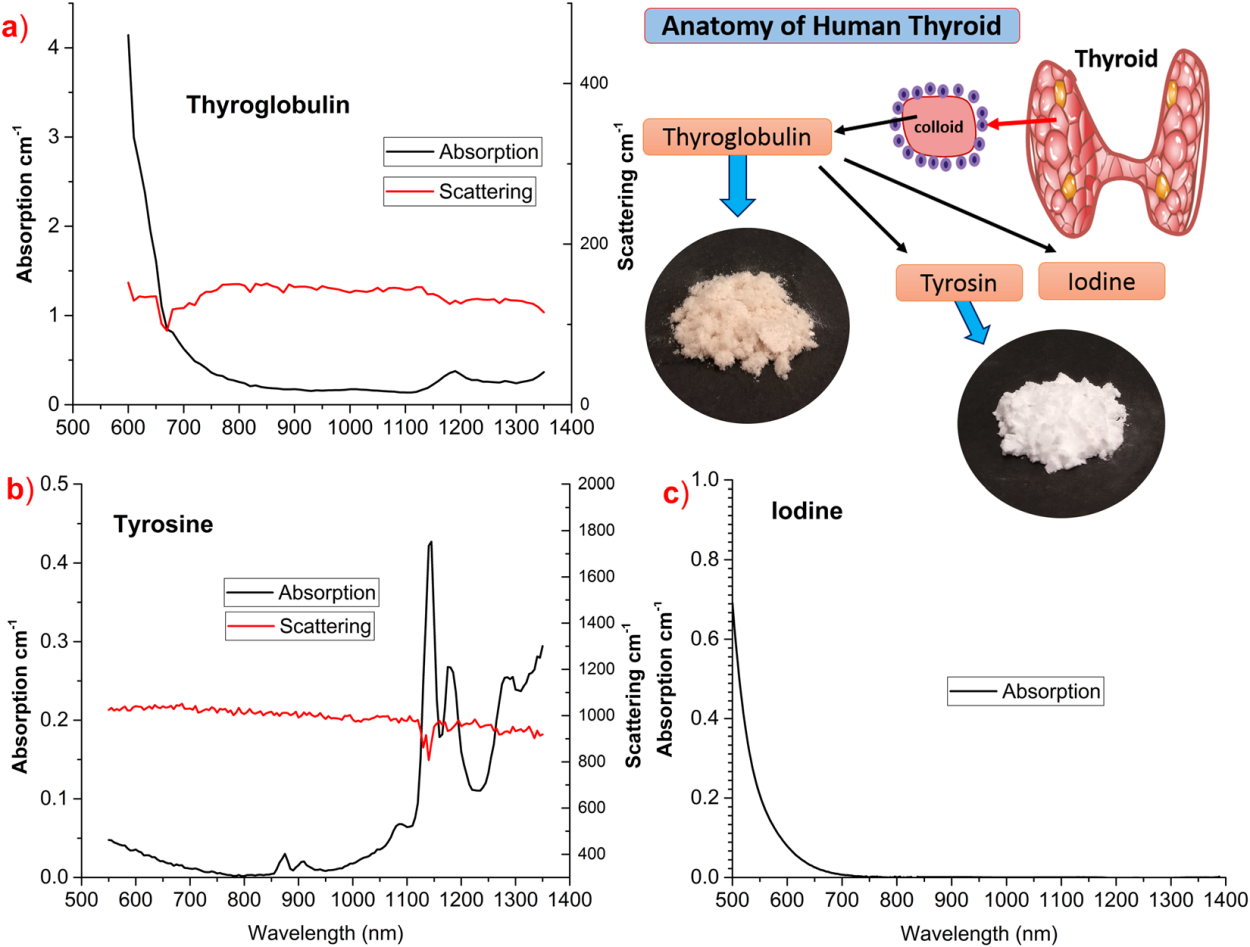
Absorption and reduced scattering spectra of (**a**) thyroglobulin, with high absorption in the visible region, and (**b**) tyrosine, with multiple peaks over the measured window; and absorption spectrum of (**c**) iodine, rapidly decreasing upon increasing wavelength reaches asymptotically zero around 800 nm. (**d**) Anatomy of the thyroid along with images of thyroglobulin and tyrosine samples are shown in insets (top right corner).

To address these limitations, we have studied the thyroid organ and identified three relevant absorbers to characterize, namely thyroglobulin, tyrosine, and iodine. Briefly, thyroid is composed by microscopic spherical sacs called thyroid follicles. The wall of each follicle consists mainly of follicular cells that produce two thyroid hormones, thyroxine or T4 and triiodothyronine or T3, which contain four or three iodine atoms, respectively. The colloid occupies the interior of the follicle and is formed mainly by thyroglobulin, a protein produced by the follicular cells that serves as a precursor for the synthesis of thyroid hormones^21,22^. A pictorial description of thyroid anatomy is shown in insets of Fig. 1. Importantly, thyroglobulin protein concentration in thyroid is relatively high: studies on the rat thyroid reveal it to be around 10–20%^22,23^.

Along with other tissue absorbers like lipid, water, collagen, oxy, deoxy- hemoglobin, considering also the specific constituents of the thyroid organ–especially thyroglobulin, tyrosine, and iodine–can lead to new resources in the pathological diagnosis of thyroid tissue.

To the best of our knowledge, in literature, there is the lack of thyroglobulin and tyrosine absorption spectra in the visible-NIR region. We have performed a preliminary work reported in reference^6^ and, based on the generally positive results obtained and critical issues identified there, we have performed the full study reported here for the characterization of tissue constituents. Unlike transparent samples, the highly diffusing nature and fluorescence of these materials in the visible region make their characterization impossible with traditional spectrophotometers. A spectrometer based on an integrating sphere^24^ can be a good option but may fail to tackle florescence and the distortion of the spectrum due to the high scattering of the samples. Diffuse optical techniques can be an optimal option to identify and eliminate distortion caused by scattering on the measured absorption spectrum of the sample^25,26^. In particular, time domain diffuse optics can naturally disentangle the absorption from the scattering coefficient^25,27^. Also the effect of fluorescence, the finite size of sample and the finite bandwidth of laser can be tackled effectively by using appropriate diffuse optical strategies^28^.

In this work, we present the absorption spectrum of tyrosine and thyroglobulin, in the 550–1350 and 600–1350 nm range, respectively. We exploited time domain diffuse optical spectroscopy (TDDOS) methods to address the above-mentioned challenges. A hybrid clinical time-resolved diffuse optical spectrometer^29^ was used for measuring highly scattering thyroglobulin and tyrosine samples, while iodine was characterized using a conventional spectrophotometer. Specific methods employed to extract absolute absorption spectra of tissue constituents and the distortion caused in their absence were discussed. A brief overview and a comparison between characterized and already known tissue constituents is presented.

## Results and Discussion

### Absorption and scattering spectra of new tissue constituents

The absorption spectra of thyroglobulin and tyrosine derived using the experimental approaches described in the Methods section are shown in Fig. 1(a,b). The spectrum of tyrosine is measured from 550–1350 nm, whereas the thyroglobulin spectrum is measured only from 600–1350 nm, due to its high absorption below 600 nm leading to strong signal attenuation. There are markedly different spectral features in the two constituent spectra. Tyrosine absorption shows six peaks at different wavelengths (875 nm, 915 nm, 1090 nm, 1145 nm, 1185 nm, 1290 nm) within the broadband (600–1350 nm) window considered, while it is negligible around 800 nm. Thyroglobulin is found to have a smoothly decreasing absorption spectrum with a shoulder around 1000 nm followed by a major peak at 1190 nm. The density of thyroglobulin (granular particles) in the sample cylinder is comparatively low with respect to tyrosine (fine powder). In spite of its low density, thyroglobulin was found to have a higher absorption as compared to tyrosine, which makes it easier to detect at low concentrations. The iodine spectrum acquired through a standard spectrophotometer is shown in Fig. 1(c). iodine seems to contribute only in the visible region: following an exponential decrease, it reaches asymptotic zero absorption around 800 nm and the absorption remains negligible up to 1350 nm. Overall, in comparison, the three tissue constituents exhibit different spectral features, which minimize coupling between them in their quantification, thus enabling their accurate estimation in clinical studies of thyroid where all three coexist.

From Fig. 1(a,b), the reduced scattering spectra of thyroglobulin and tyrosine were found to be rather flat or only slightly decreasing with wavelength, with high mean values of 136 and 990 cm^−1^, respectively. A minor bandwidth effect due to the sharp change in the absorption spectrum of tyrosine at 1145 nm is evident in the form of a dip in its reduced scattering spectrum^30^. The fine powder form of tyrosine is reflected in its very high scattering coefficient.

As displayed in Fig. 1(c), the absorption of iodine decreases rapidly upon increasing wavelengths and no spectral feature is observed. Povidone-iodine (PVP-I) solution, which is a chemical complex of iodine, is a transparent solution (*i.e*., with negligible scattering), and a commercial spectrophotometer was used to obtain the iodine spectrum. The spectrum obtained from the spectrometer was verified with the spectrum obtained by diffuse optics (as explained in the Methods section). We found good agreement between the spectra measured with the two techniques.

### Challenges in the extraction of the absorption spectra

The retrieval of the absorption spectra of tyrosine and thyroglobulin set some challenging experimental difficulties related to scattering, fluorescence, boundary effects, and bandwidth effects, as detailed below.

**Figure 2:**
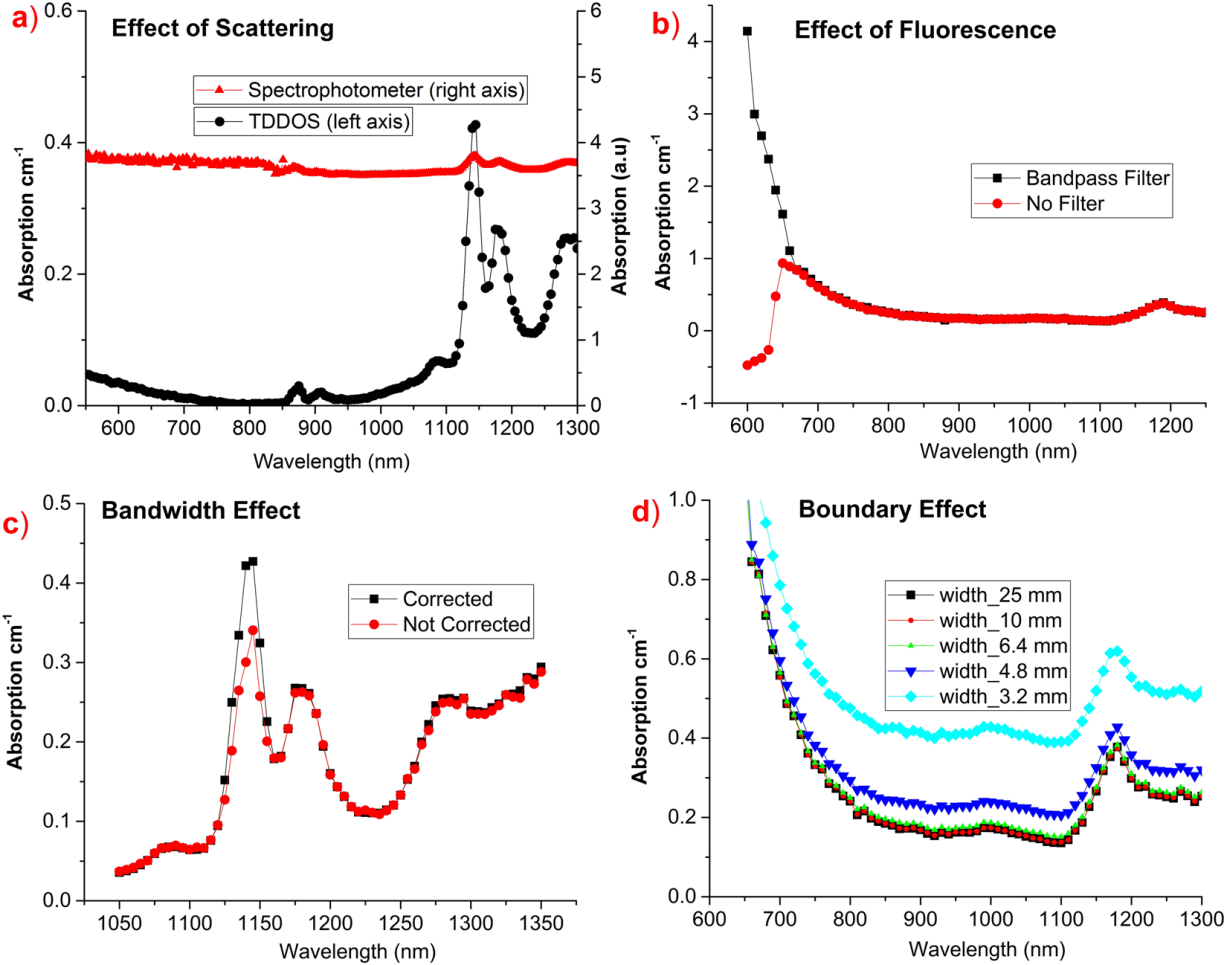
Comparison of absolution absorption spectrum with various distortion effects: (**a**) absorption features distorted by high scattering of tyrosine (red triangles), (**b**) fluorescence distorting the spectrum of thyroglobulin in the visible region (red circles), (**c**) Effect of finite laser bandwidth at sharp absorption change of tyrosine (1145 nm peak, red circles), (**d**) overestimation of absorption at small sample sizes (sample width: 3.2 mm, 4.8 mm, 6.4 mm), only spectra above 10 mm sample width match with each other.

Pure tyrosine and thyroglobulin are available in powder form, therefore standard spectrophotometer extinction spectra are severely affected by scattering contributions. A comparison between the tyrosine spectra obtained using a commercial spectrometer and our time domain system is shown in Fig. 2(a). Clearly, the adoption of the time domain scheme permitted to disentangle absorption from scattering contributions whereas the spectrophotometer spectrum is almost completely dominated by the very high scattering of the samples (Fig. 1(b)). Furthermore, to reduce the noise in the retrieved absorption spectrum, we have applied a method well described elsewhere^31,32^, which allowed us to avoid the residual coupling of the absorption and scattering coefficient due to the limitations of the Diffusion approximation with also improved smoothness of the spectrum. First, the absorption and scattering spectra were retrieved, as described in the Methods section, then, the scattering spectrum was fitted using a power law derived from Mie theory and used as *a priori* information in the fitting procedure to derive an absorption spectrum less prone to fitting instabilities.

The presence of fluorescence was observed for thyroglobulin in the visible range (600–700 nm). Fluorescence affects the tail of the temporal curves, reducing their slope and thereby leading to underestimated absorption values. This effect was eliminated by means of bandpass filters (bandwidth 10 nm). In the absence of filters, a relative error of 107% in absorption estimation was observed over the 600–650 nm range, as shown in Fig. 2(b). Additionally, as described in the Methods section, fitting was performed over different fitting ranges to understand the effectiveness of filters in suppressing fluorescence photons. All fitting ranges yielded similar results, thus reconfirming the elimination of fluorescence effects.

In the reduced scattering spectrum of tyrosine, a slight laser bandwidth effect is observed at 1145 nm. In general, the bandwidth effect becomes evident in diffuse optics when a large bandwidth (>5 nm) source encounters sharp absorption changes^30^, leading to a deformation of the photon temporal distribution. In practice, the crosstalk between high absorbed and low absorbed photons due to large laser bandwidth increases with the photon time-of-flight, which in turn, affects the tail of the temporal curve leading to absorption underestimation. To effectively eliminate this perturbation, data analysis was carried out taking the spectral bandwidth of the laser into account at each wavelength. In this method, a weighted average of the laser bandwidth contribution was considered to generate theoretical light diffusion curves, which were used to fit the measured time distributions, thereby effectively nullifying the effect of the laser bandwidth on the estimated absorption spectrum^30^. The only assumption is a constant scattering coefficient over the considered laser bandwidth, which is a reasonable approximation considering the limited spectral range considered and the flat behavior of the reduced scattering spectrum. Figure 2(c) shows the distortion caused to the tyrosine peak (1145 nm) in the absence of bandwidth correction, displaying a relative error of 28% in the absence of correction.

Boundary effects must be taken into account because of the limited sample volume, as they scale with decrease in scattering and size of the sample volume. Fortunately, both thyroglobulin and tyrosine exhibit very high scattering and the sample holder is 25 mm in width, making measurements less prone to boundary effects. However, to understand the limiting point we performed simulations with optical properties (absorption and reduced scattering) of thyroglobulin (more vulnerable due to lower scattering) in a sample box of different widths (3.2, 4.8, 6.4, 10, 25 mm), which has been fitted using the semi-infinite model. It is evident from Fig. 2(d) that the absorption is incrementally overestimated for widths less than 10 mm. Hence, the limiting point is around 10 mm, whereas the sample holder used for the measurement has width of 25 mm, which is more than twice as compared to the limiting point, reaffirming the absence of boundary effects in the optical properties extraction.

### Comparison with other tissues constituents

**Figure 3:**
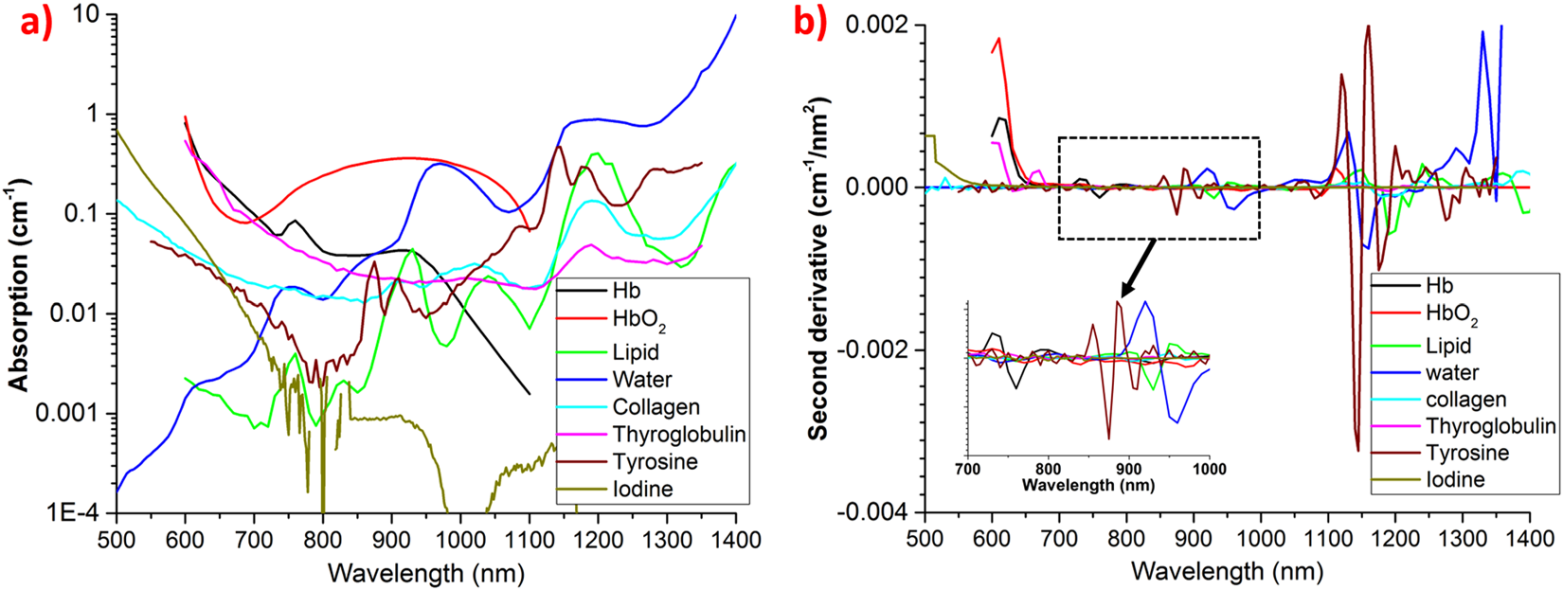
Comparison of thyroglobulin, tyrosine and iodine spectra with typical tissue constituent spectra (oxy- and deoxy-hemoglobin, lipid, water, collagen): (**a**) tissue constituent spectra with absorption values relevant to thyroid tissues^6^, (**b**) second order derivative spectra of various tissue constituents and the inset reveals some unique features of tyrosine spectrum.

Though tyrosine and thyroglobulin have various interesting absorption peaks, it is hard to judge on the key peak or region for their quantification, unless they are compared with the other important tissue constituents. Figure 3(a) plots the new thyroid tissue constituents measured by our studies against already known typical tissue absorbers, namely: deoxy-hemoglobin (Hb), oxy-hemoglobin (HbO_2_), lipid, water, collagen. The concentration values for the spectra shown in Fig. 3 were obtained from mean values of tissue constituents in the thyroid location of reference^6^. Though the reference^6^ used homogenous model for constituents estimation (values contaminated by superficial tissue), it could be considered as preliminary values for thyroid constituents estimation. To enable easy differentiation among different tissue constituents the second order derivation spectra are shown in Fig. 3(b). Tyrosine has many unique features for its quantification. Especially, the tiny peak at 875 nm is unique to tyrosine which is evident from the derivative spectra (inset in Fig. 3(b)). However, its low absorption might pose challenges for its quantification. The sharp change in absorption features of tyrosine over the entire broadband region is evident from derivative spectra with maximum swing around 1145 nm peak. The peak around 1090 nm is interesting, as it is intense and overlaps with local minima of water, lipid and collagen absorption, making it an ideal location for tyrosine quantification. Though tyrosine has an increasing absorption around 600 nm range, high absorption dominance of Hb, HbO_2_, Thyroglobulin can make it a weak location for tyrosine detection.

In case of thyroglobulin, there is the associated risk for its coupling with collagen, due to similarities in their spectral features. With careful analysis, it can be seen that collagen shows peaks at 930 nm and around 1030 nm that are absent in the thyroglobulin spectrum. Hence, at least a point in one of these regions is needed to allow distinguishability between these chromophores. Specifically, a point around 1000 nm is more beneficial, as it falls at the intermediate point of water and lipid peaks around 1000 nm. The steeper change in thyroglobulin absorption around 600 nm can also be considered as a good feature for its extraction. As it is observed from both Fig. 3(a,b), thyroglobulin has absorption similar to Hb and HbO_2_, and could be distinguished with sufficient signal to noise ratio in an *in vivo* scenario. Attention should be paid to avoid coupling between the similar features of hemoglobin spectra. However, a peak (660 nm) in the derivative spectrum of thyroglobulin is a unique feature, which is absent in Hb and HbO_2_ spectra and might thus help in avoiding coupling. The effects of other chromophores, such as Cytochrome C Oxidase (CCO), elastin can be studied using this methodology in the future if *in vivo* follow-up studies reveal their signatures in the thyroid. In summary, both thyroglobulin and tyrosine have features distinguishable as compared to other tissue constituent spectra. Apart from the *in vivo* application discussed above, the spectra presented here can also be exploited for *in vitro or interoperative* optical characterization of thyroid tissue.

## Conclusion

To the best of our knowledge, we have presented for the first time the absorption spectra of tyrosine and thyroglobulin–in powder and granular form–which are tissue constituents specific to the thyroid organ, in the 550 to 1350 nm range. We also characterized the iodine spectrum using a standard spectrophotometer. The measurements and data analysis were performed rigorously by answering various challenges posed by the highly scattering samples. The effect of fluorescence was suppressed by means of bandpass filters, which eliminated an error of 107% in the 600–650 nm range. A bandwidth compensation algorithm effectively made up for the effect of the laser bandwidth that induced 28% error on the tyrosine peak at 1145 nm. The boundary effects due to the finite size of the sample volume were found to be negligible adopting the geometry used in this study. Considerable difference is observed among the measured spectra of the thyroid constituents enabling easy distinction between them.

To answer the relevance of thyroid specific absorption spectra to *in vivo* studies, a brief comparison was presented between our newly characterized thyroid absorbers and constituents (hemoglobin, lipid, water, collagen), which are typical of human tissues. Importantly, key spectral regions that are significant for the *in vivo* detection of tyrosine and thyroglobulin were proposed and will be tested as the next step in our studies. The path towards the *in vivo* optical quantification of tyrosine and thyroglobulin is still an open and challenging issue. One could imagine that the spectral contribution could be hindered by the strong absorption of other tissue constituents (e.g. hemoglobin in the visible range, water in the NIR range). Also, the *in vivo* environment (pH, aggregation state) could definitely alter the absorption spectrum as compared to the one obtained here on powder samples. This is a topic for a further interesting investigation, which could open new opportunities for the diffuse optical diagnosis of thyroid related pathologies.

## Methods

### Time-resolved diffuse optical instrumentation

**Figure 4:**
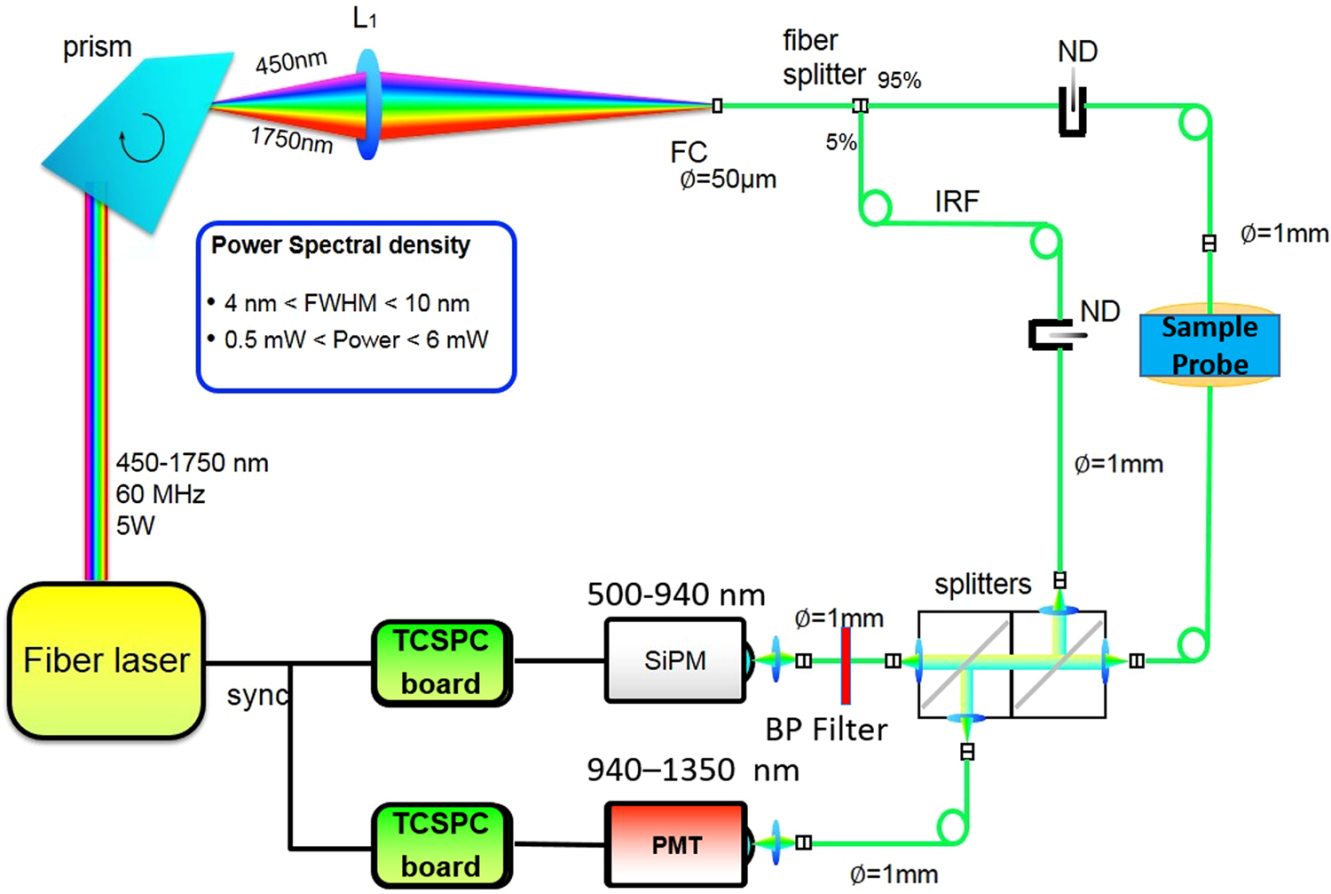
Optical layout of time resolved diffuse optical instrumentation. The two detectors effectively cover the broadband range (500–1350 nm).

A schematic layout of the system is shown in Fig. 4. The system is powered by a supercontinuum pulsed fiber laser (450–1750 nm) operated at 60 MHz repetition rate. Wavelength tunability is achieved by the Pellin Broca prism, which couples the selected wavelength into a 50 µm core source fiber. Then, the laser beam is split into two parts of 95% and 5% power: the former acts as source to the sample, whereas the latter is the reference arm, which makes the system immune to drift and distortions caused by the laser and environment. The setup employs two detectors to provide high responsivity and signal to noise ratio over the entire measurement window (500–1350 nm): specifically, in the 500–940 nm range a Silicon Photomultiplier (SiPM, Excelitas Technologies, C30742-11-050-T1) and in the 940–1350 range an InGaAs photomultiplier (Hamamatsu H10330A-45). More details on the system and its validation can be found elsewhere^29,33^.

The broadband diffuse optical system used for this study was extensively characterized following the MEDPHOT Protocol^34^ in terms of accuracy, linearity, stability and reproducibility^29^. In brief, the accuracy of the system was validated against the well-known spectrum of water, demonstrating good agreement in the 600–1350 nm region, spanning almost 3 decades of μ_a_ values (from 0.002 up to 1.2 cm^−1^). Under standard operating conditions (10^6^ photon count rate, μ_a_ = 0.1 cm^−1^, µ_s_’ = 10 cm^−1^), the noise for the absorption retrieval was found to be less than 2% (coefficient of variation). The stability over 10 hours and day to day reproducibility were found to be less than 3% and 4% (coefficient of variation), respectively.

### Measurement protocol and data analysis

The L-tyrosine and thyroglobulin needed for our study were procured from Sigma Aldrich (part no. 93829 and T1126, respectively). L-tyrosine is a white fine powder, whereas thyroglobulin (extracted from porcine thyroid) is more granular with ivory complexion. More details on certificate of analysis and certificate of origin can be found on Sigma website under lot no. BCBP3212V and 018K7012V for tyrosine and thyroglobulin, respectively. The fine size of both samples allows effective use of diffuse optical techniques as it increases scattering and reduces boundary effects. For measurement, the samples were placed in a sample holder which is a cylinder of 25 mm diameter. The density of tyrosine (0.320 g/cm^3^) in the cylinder is comparatively higher than that of thyroglobulin (0.219 g/cm^3^). Measurements were performed in transmittance geometry with 2.2 mm and 3.2 mm thickness (source-detector separation) for tyrosine and thyroglobulin, respectively. Measurements were repeated four times over 600–1350 nm at steps size of 5 nm and 10 nm, respectively, for tyrosine and thyroglobulin.

In case of iodine, as it is a transparent liquid, we used a standard spectrophotometer (JASCO V-570) to assess the spectrum over 500–1350 nm. Povidone-iodine (PVP-I) which has 10% iodine was procured from pharmacy (pharmaiodio). The PVP-I was diluted in water at 0.8% solid fraction and 1 cm quartz cuvette was used for the measurements. For comparison, PVP-I absorption was measured also using diffuse optical methods. For this purpose, a mixture of iodine, water and Intralipid® (to increase scattering) was made with 1% solid fraction of Intralipid (estimated reduced scattering coefficient 10 cm^−1^). The above described diffused optical spectrometer was exploited to retrieve the iodine spectrum from the aqueous solution of Intralipid®.

The optical properties of the samples were extracted by fitting the measured temporal curves to the solution of the semi-infinite slab model^35^ of the diffusion equation with extrapolated boundary conditions^36^ after convolving with the Instrument Response Function. The temporal curve was effectively utilized for fitting the measured time-resolved curves from 80% of the rising edge down to 1% of the falling edge for residual minimization.

We have found that thyroglobulin exhibits fluorescence in the visible region (600–700 nm). Thus, bandpass filters (BP filter) of 10 nm bandwidth were used in front of the visible range detector at these wavelengths to prevent fluorescence light from entering the detector. In general, due to long decay times, fluorescence photons affect the tail of the temporal curves. The effectiveness of filters to eliminate fluorescence was tested by performing data analysis at different fitting ranges (80% to 50%, 80% to 10%, and 80% to 1%); with and without photons from the tail of the temporal curves. In the absence of fluorescence photos data analysis at different fitting range will yield same absorption and scattering spectra. The distortions caused by the finite bandwidth of the laser was eliminated by accounting for it in the data analysis^30^. The effect of boundaries on the absorption spectrum due to finite width of the sample cylinder was thoroughly studied to understand the limiting width of sample probe for the optical properties of the samples considered in this study.
